# BIOMARKER STRATEGIES FOR GEROSCIENCE-GUIDED CLINICAL TRIALS

**DOI:** 10.1093/geroni/igz038.2731

**Published:** 2019-11-08

**Authors:** Jamie N Justice, George A Kuchel, Nir Barzilai, Stephen Kritchevsky

**Affiliations:** 1 Wake Forest School of Medicine, Winston-Salem, North Carolina, United States; 2 university of connecticut, Farmington, Connecticut, United States; 3 Albert Einstein College of Medicine, Bronx, New York, United States

## Abstract

Significant progress in the biology of aging and animal models supports the geroscience hypothesis: by targeting biological aging the onset of age-related diseases can be delayed. Geroscience investigators will test this hypothesis in a multicenter clinical trial, to determine if interventions on biological aging processes can prevent accumulation of multiple age-related diseases and aging phenotypes in older adults. Prodigious activity is underway to develop markers of biological aging, but currently there is no aging biomarker consensus to support geroscience-guided clinical trial outcomes. We convened an expert committee to establish a framework for selection of blood-based biomarkers, emphasizing: feasibility/reliability; aging relevance; ability to predict clinical trial outcomes; and responsiveness to intervention. We applied this framework and identified a short-list of blood-based biomarkers with potential use in multicenter trials on aging. We review progress on efforts to test these candidate biomarkers of aging and development of biomarkers strategy for geroscience-guided clinical trials.

